# *Mycobacterium avium* Subspecies *paratuberculosis* DNA and Antibodies in Dairy Goat Colostrum and Milk

**DOI:** 10.3390/vetsci6040096

**Published:** 2019-11-29

**Authors:** Karianne Lievaart-Peterson, Saskia Luttikholt, Maaike Gonggrijp, Robin Ruuls, Lars Ravesloot, Ad P. Koets

**Affiliations:** 1 Department of Small Ruminant Health, GD Animal Health, P.O. Box 9, 7400 AA Deventer, The Netherlands; s.luttikholt@gdanimalhealth.com; 2 Epidemiology Group, GD Animal Health, P.O. Box 9, 7400 AA Deventer, The Netherlands; m.gonggrijp@gdanimalhealth.com; 3 Department of Bacteriology and Epidemiology, Wageningen Bioveterinary Research, 8221 RA Lelystad, The Netherlands; robin.ruuls@wur.nl (R.R.); lars.ravesloot@wur.nl (L.R.); ad.koets@wur.nl (A.P.K.); 4 Department of Farm Animal Health, Faculty of Veterinary Medicine, Utrecht University, 3584 CL Utrecht, The Netherlands

**Keywords:** *Mycobacterium avium* subspecies *paratuberculosis*, goat, colostrum and milk

## Abstract

*Mycobacterium avium* subspecies *paratuberculosis* (MAP) is endemic in the Dutch dairy goat population causing economic loss, and negatively influencing welfare. Moreover, there are concerns about a potential zoonotic risk. Therefore the industry’s objectives are to decrease MAP prevalence, limit economic losses as well as reduce the concentration of MAP in (bulk) milk. To diminish within-farm spread of infection, vaccination, age dependent group housing with separation of newborns from adults, as well as rearing on artificial or treated colostrum and milk replacers are implemented. However, the importance of MAP contaminated colostrum and milk as a route of infection in dairy goat herds is unknown. Therefore the aim of this study was to detect the presence of MAP DNA in colostrum and milk from dairy goats in infected herds. A convenience sample of 120 colostrum samples and 202 milk samples from MAP infected dairy goat herds were tested by IS900 real-time Polymerase Chain Reaction (PCR) for MAP DNA. Furthermore, 22 colostrum samples and 27 post mortem milk samples of goats with clinical signs consistent with paratuberculosis from known infected herds were tested. The majority of samples were from goats vaccinated against MAP. Positive or doubtful PCR results were obtained in none of the 120 and two of the 22 colostrum samples, and in eight of the 202 and four of the 27 milk samples Negative PCR results were obtained in the remaining 140 (99%) colostrum samples and 217 (95%) milk samples.

## 1. Introduction

*Mycobacterium avium* subspecies *paratuberculosis* MAP infection causes a regional intestinal inflammation resulting in paratuberculosis (Johne’s disease) in domestic and wild ruminant species worldwide. Subsequently, MAP-infection causes a protein loosing enteropathy resulting in loss of body condition, dry and flaky skins, poor hair or wool condition, edema, and decreased productivity [1,2]. Cattle and sheep show intermittent diarrhea, which is very seldom seen in goats. It is a major health and welfare issue, and can cause severe economic losses [3,4]. This disease is presenting more or less different in goats than in sheep or cattle [5], although MAP transmission is thought to follow similar pathways. The fecal-oral route including drinking contaminated colostrum or milk is considered the most important [6].

Although a causal role between MAP and Crohn’s disease in humans has not been definitively established [7], a proactive approach in ensuring consumer confidence by addressing the issue is sensible [8,9]. Therefore, MAP control programs have been initiated in (at least) 22 countries [10].

The Dutch dairy cattle and goat industries objectives are to decrease MAP infection prevalence, limit farm-level economic loses, as well as reduce MAP load in (bulk) milk [10,11,12,13]. Elimination of MAP-infection, such as achieved in the Norwegian goat population [14], currently seems to be a bridge too far. Herd level prevalence of exposure to MAP in Dutch dairy goat herds is estimated at 78% based on clinical and routine (patho)diagnostic observations [15]. Dairy goat farmers are increasingly motivated to implement strategic measures to reduce MAP transmission because of an evolving concern about the quality and sales of dairy products and to limit economic loss. For example, vaccination against MAP is commonly used in Dutch dairy goat herds.

MAP is assumed to be transmitted via colostrum and milk. In cattle colostrum and milk contamination with MAP through fecal contamination of teats or shedding from within the udder has been demonstrated [16,17,18]. Thus, in the Netherlands it is common practice to snatch goat kids at birth, house them separately in age groups, and feed them cow or artificial colostrum and milk replacers to reduce transmission of MAP, Caprine Arthritis Encephalitis Virus (CAEV) and Caseus Lymphadenitis (CLA). However, feeding cow or artificial colostrum increases the risk of failure of passive transfer of maternal antibodies, which in turn leads to increased morbidity and mortality from infectious disease in young goats [19]. Commercially available colostrum replacers have proven to be inadequate substitutes for goat colostrum as a source of gamma globulins [20], and even colostrum substitute derived from goat serum resulted in lower serum IgG concentration [21]. Anecdotally, the benefits of goat colostrum improves rearing results by reducing mortality and increasing growth rate and improves resilience to disease in Dutch dairy goat herds. A large proportion of the Dutch dairy goat herds are CAEV and CLA certified free (GD-Animal Health). In these herds, MAP is the main reason not to use goat colostrum. However, there is only limited data on the excretion of MAP in colostrum and milk in dairy goats. Therefore, the aim of this study was to detect the presence of MAP DNA in colostrum and milk from dairy goats in infected herds.

## 2. Materials and Methods

### 2.1. Collection of Samples

Initially, it was intended to collect colostrum and milk samples from dairy goat herds with a history of paratuberculosis cases where no vaccination against paratuberculosis had been applied. However, given the widespread use of vaccination, this showed unfeasible. Thus, in addition to one unvaccinated herd (C_nv_), four herds (A, B, D, E) in which goats were vaccinated with a licensed paratuberculosis vaccine Gudair^®^ (CZ Veterinaria SA, Porriño, Spain) as well as one herd (F_pv_) that had partially purchased vaccinated goats were included in the study. In addition, colostrum from the milk of MAP-ELISA (antibody)-positive goats from a seventh herd (I) was included. A further two herds (G and H), in addition to one of the above (B), sent in suspected paratuberculosis goats for post mortem examination. Each of these nine herds had a history of paratuberculosis cases that had been confirmed by laboratory testing in the previous years (Table 1).

Four sets (I-IV) of colostrum and milk samples were collected:

(I) Colostrum samples (n = 120) were collected from a convenience sample of dairy goats from each of the six dairy goat herds. It was intended to collect at least 150 samples, as this would allow to conclude with a level of confidence of 95% that the true proportion contaminated samples did not exceed a designMAP prevalence of 2% if none of the samples tested MAP positive. Preferably between 20 to 30 goats per herd were sampled by the farmer, of which 10 were one year old first lactation goats and the rest a representative representation of ages and/or lactations were collected as per written instruction. Samples were collected in provided vials, stored in the fridge, and picked up on farm by the first author for cooled transport to the GD-Animal Health laboratory.
(II) Colostrum samples (n = 22) were collected from a convenience sample of ELISA-positive dairy goats with clinical signs consistent with paratuberculosis from two dairy goat herds (herds A and I). These samples were collected by the farmers as described above.
(III) Milk samples (n = 202) from a convenience sampling of dairy goats from a single herd (herd B). These samples were collected by the farmers as described above.
(IV) Post mortem collected milk samples (n = 27) from goats designated to be slaughtered from three herds (herd B, G, and H). These goats were selected by the dairy goat farmers based on clinical signs consistent with paratuberculosis and were euthanized on farm by the local veterinarian. After euthanisation, the cadavers were transported to the post mortem facility at GD-Animal Health. Milk samples were collected by milking both udder halves separately after disinfection and removal of the first milk. In addition, tissue samples were collected from the distal ileum, mid jejunum, ileocecal valve, draining mesenteric lymph nodes, as well as bilateral mammary tissue, and mammary lymph nodes. For each individual, four pooled tissue samples were created, consisting of samples from the intestines (distal ileum, mid jejunum, ileocecal valve), the draining mesenteric lymph nodes, bilateral mammary tissue, and the bilateral mammary lymph nodes, respectively.

### 2.2. Laboratory Analyses

All colostrum and milk samples were tested for both antibodies against MAP as well as MAP DNA. All pooled tissue samples were tested for MAP DNA.

Antibodies were detected by the ID Screen Paratuberculosis Indirect Screening Test (ID Vet, Montpellier, France). The test is an *M. phlei* absorbed Enzyme-Linked Immuno Sorbent Assay (ELISA) detecting anti-**MAP** IgG adapted to colostrum, and milk testing as described in literature [15]. Sample-to-positive ratio’s (%S/P) <15% were considered negative, 15–30% doubtful, and >30% positive.

For direct MAP detection, samples were pre-processed for DNA extraction, and subsequently tested by IS900 PCR. The milk and colostrum samples were homogenized on a roller bank for 10 min. From the homogenized colostrum and milk, a 2 mL sample was collected in an Eppendorf 2 mL tube. The samples were centrifuged at 4 °C for 10 min at 10,000 × *g*. The cream and milk serum fractions were carefully removed. The pellet was resuspended in 200 µL phosphate buffered saline (PBS) prior to DNA isolation. Tissues (approximately 3 g per sample) were mechanically homogenized using bead beating (Ultra Turrax^®^ tube and tube drive workstation, IKA, Staufen, Germany) for 90 s at 6000 rpm in 10 mL of phosphate buffered saline (PBS). A sample of 200 µL colostrum, milk, and tissue homogenate was subsequently processed for DNA isolation using the DNeasy blood and tissue kit (Qiagen Benelux, Venlo, The Netherlands) following instructions provided by the manufacturer. Technical details of the IS900 PCR have been published previously [22]. In short, the IS900 PCR was run on an Applied Biosystems 7500 Fast Real-time PCR system (Thermo Fisher Scientific, Landsmeer, The Netherlands). The PCR was performed using PerfeCTa Multiplex qPCR supermix UNG (VWR International B.V., Amsterdam, The Netherlands), IS900 F forward primer 5′-CCGCTAATTGAGAGATGCGATT-3′ (400 nM), IS900 Probe 5′-6FAM–ACCTCCGTAACCGTCATTGTCCAGATCA-BHQ1-3′ (200 nM), IS900 R reverse primer 5′-CCAGACAGGTTGTGCCACAA-3′ (400 nM) and IS900 IPC inhibition control probe 5′-YY-CTGCTGGGTATACGTCGTCTAAGTCCGAATC-BHQ1-3′ (200 nM) in a reaction volume of 20 µL (all primers and probes from Integrated DNA Technologies, Leuven, Belgium). The template DNA consisted of 2 µL of the eluate of the isolated tissue DNA and 1 µL of inhibition control DNA (constructed by designing a non-sense DNA sequence with similar GC content compared to the IS900 amplicon, containing the probe site and flanked by the IS900 primer binding sites). In each PCR run, a negative (nuclease free water) control and a positive (MAP strain B854 genomic DNA) control were included. The reaction conditions were 5′ at 45 °C, 1′ at 95 °C, followed by 50 cycles of 10′’ at 95 °C, and 30′’ at 60 °C. Data were analysed using the 7500 Fast System SDS software to determine Ct values and check for inhibition to flag potential false negative results. When inhibition was detected samples were rerun at a 1:10 template dilution. Samples with Ct-values ≤36 were considered positive, Ct-values between 36 and 45 were considered doubtful, and Ct-values ≥45 were considered negative.

### 2.3. Data Analyses

All statistical analyses were carried out in STATA 15.0 (StataCorp, College Station, TX, USA, 2017). The effect of farm, vaccination, time post vaccination, and age at vaccination on S/P ratio was assessed by a one-way ANOVA with a post hoc Dunn’s multiple comparisons test.

### 2.4. Animal Welfare and Ethical Considerations

All participating goats were assumed to be farmed in accordance with national or EU laws and regulation. Since the collection of colostrum and milk from dairy goats, as well as euthanasia of goats designated for slaughter are not deemed as invasive or painful or stressful, but rather standard agricultural practice, the institutional Animal Welfare Body of GD Animal Health (*AWB Advice regarding EU/2010/63 art. 5*) ruled that this study did not require approval from an independent Animal Ethics and Care Committee.

## 3. Results

### 3.1. Colostrum

MAP DNA was detected in none of the 120 colostrum samples from sample set I; all PCR results were negative. Given that less than the intended 150 samples were collected, it was not possible to conclude with 95% certainty that the prevalence of shedding of MAP in colostrum was lower than the design prevalence of 2%. Assuming that these samples were representative for the total population of dairy goats on farms with a history of paratuberculosis cases, the 95% CI of the true proportion of dairy goats shedding MAP in colostrum could be estimated between 0% and 2.4%. However, two of the 22 colostrum (9%, 95% CI: 1–29%) samples from ELISA-positive goats with clinical signs of paratuberculosis (sample set II) yielded a dubious PCR result.

**Figure 1:**
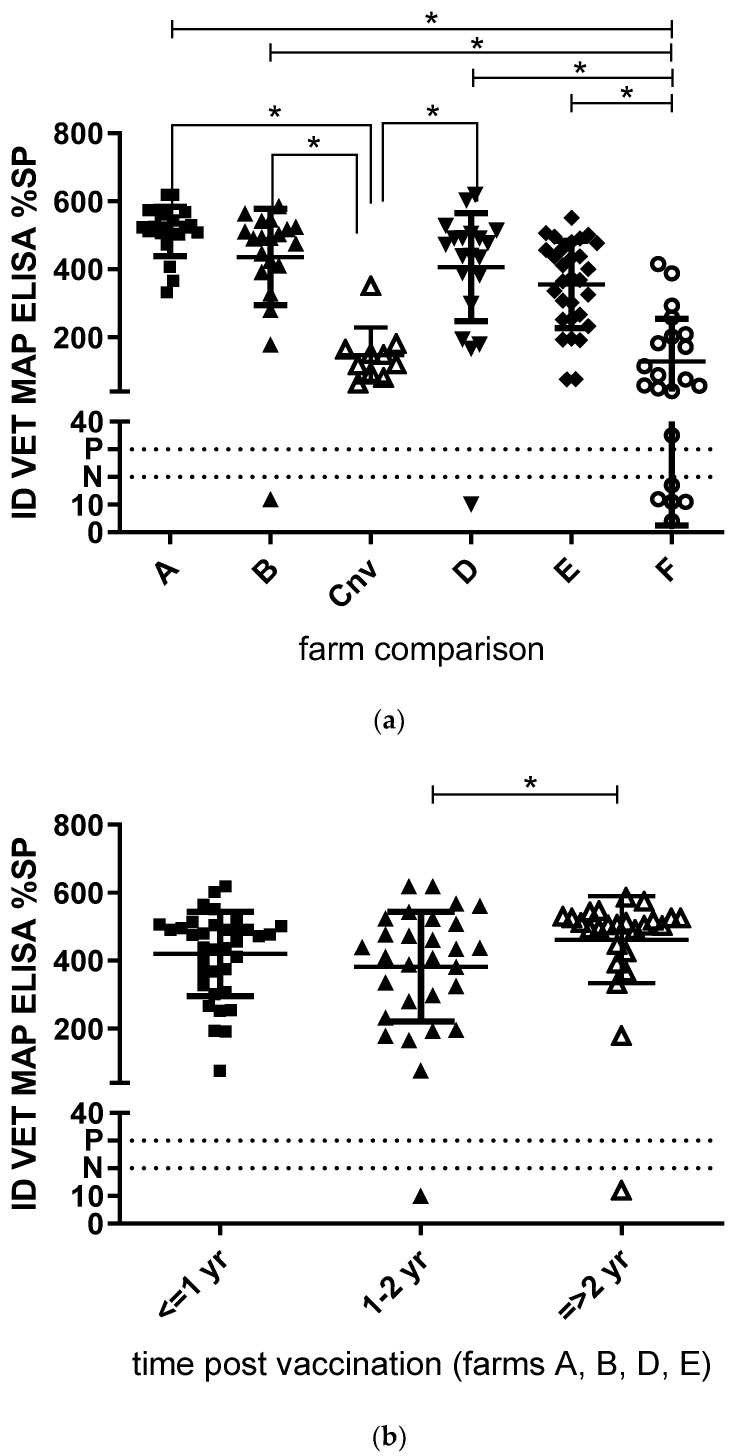

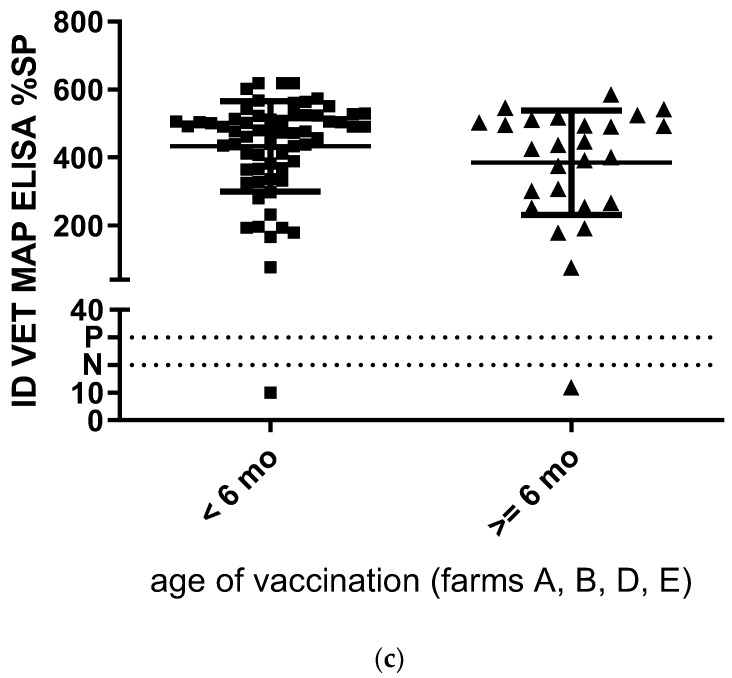
Comparison of paratuberculosis antibodies (IDVet, MAP, ELISA) in colostrum samples. (**a**). Samples from six commercial dairy goat farms (A, B, D, E Gudair^®^ vaccinating, C non vaccinating, and F partially vaccinating. Total n = 120). (**b**). Samples from four Gudiar^®^ vaccinating commercial dairy goat farms. One year, between one and two years, and more than two years post vaccination. (**c**). Samples from four Gudiar^®^ vaccinating commercial dairy goat farms. Vaccinated as kids younger than six months of age, and older than six months of age. The population mean ± 1×SD is indicated per farm. The horizontal line at %SP 30 indicates positive cut-off; the horizontal line at %SP 20 indicates negative cut-off. Samples with values between %SP 20 and 30 are considered dubious. Kruskal–Wallis *p* < 0.0001; * Dunn’s multiple comparisons test *p* < 0.05.

Map antibodies were detected by ELISA in 114 of the 120 (95%, 95% CI: 90–98%) colostrum samples from set I and in all 22 of the 22 of the 22 colostrum samples from set II. Positive ELISA results were obtained in samples from each of the six dairy goat herds (Figure 1a), confirming exposure to MAP infection and/or vaccination in these herds.

As shown in Figure 1a, there were significant differences in %SP between three out of the four fully vaccinating (A *p* < 0.0001, B *p* < 0.01, and D *p* < 0.05) and non-vaccinating herds (Cnv), though not with the fifth herd (E). One herd had some purchased vaccinated goats within their unvaccinated herd (F), and antibody levels did not differ significantly with the non-vaccinating herd, but did with the others (A *p* < 0.0001, B *p* < 0.0001, D *p* < 0.001 and E *p* < 0.01). There was no difference in time post vaccination: less than one year, between one and two years, or more than two years ago (Figure 1b). Even so, there was no significant difference in antibodies depending on age at vaccination (as kids <6 months of age, or as young stock >6 months of age) (Figure 1c).

### 3.2. Milk

MAP DNA was detected in eight (4%; 95% CI: 2–8%) (3× positive and 5×dubious) of the 202 milk samples from set III. MAP antibodies were detected by ELISA in 96 (47%, 95% CI: 40–54%) of the 202 milk samples, confirming exposure to MAP infection and/or vaccination in herd B. There was no 100 percent one-on-one match between antibody and DNA positives as three ELISA-positives tested PCR-dubious, but of the ELISA-negatives two tested doubtful, and another two positive in the PCR (Table 2). But, more importantly, 102 ELISA-negatives tested PCR-negative and there were no PCR-positives.

### 3.3. Milk and Tissue Samples Collected Post Mortem

In two of 29 submitted goats designated to be slaughtered with clinical signs consistent with paratuberculosis (sample set IV), no milk sample could be obtained post mortem. Since milk was sampled from both udder halves, 54 milk samples from the remaining 27 goats, were available for analyses. Doubtful PCR results were obtained in seven (four goats) of the 54 milk samples. The remaining 47 samples yielded a negative PCR result.

**Figure 2:**
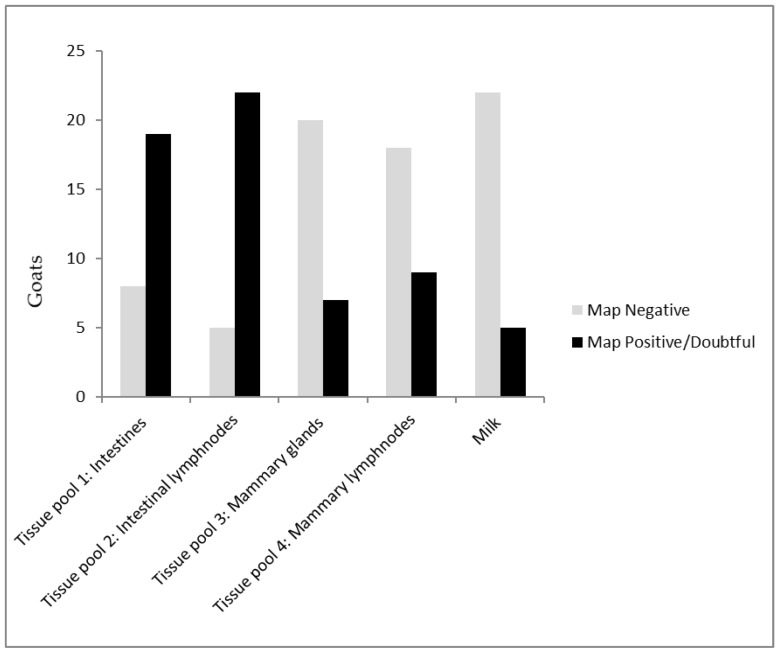
*Mycobacterium avium* subspecies *paratuberculosis* (MAP) IS900 PCR results of tissue and milk from 27 goats with clinical signs of paratuberculosis from herds with confirmed paratuberculosis cases. Tissue pool 1: distal ileum, mid jejunum, ileocecal valve. Tissue pool 2: draining mesenteric lymph nodes. Tissue pool 3: bilateral mammary tissue. Tissue pool 4: mammary lymph nodes.

**Figure 3:**
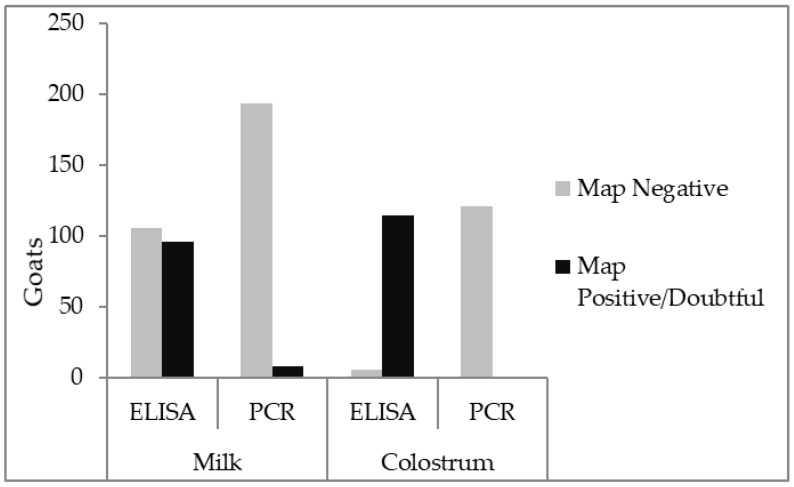
*Mycobacterium avium* subspecies *paratuberculosis* (IS900 PCR) and antibody (ID Vet MAP ELISA) results in milk (sample sets III (n = 202) and IV (n = 27), from five herds) and colostrum (sample set I (n = 120) and II (n = 22), from six herds) from goats originating from herds with paratuberculosis.

Post mortem macroscopic changes suggestive for paratuberculosis including thickening of the intestinal mucosa, lymphangitis, and lymphadenitis of draining mesenteric lymph nodes, were observed in 20 of the 27 cases. Results from the twenty seven sacrificed goats showed 19 (70%, 95% CI: 55–85%) had a positive or doubtful PCR signal in the intestines and 22 of the 27 (82%, 95% CI: 67–97%) in the mesenteric lymph nodes (Figure 2). Only within three goats both the intestines and mesenteric lymph nodes were negative in the PCR. In nine of the 27 (30%; CI: 17–47%) the mammary lymph nodes had a PCR signal as well as seven of the 27 (33%; CI: 15–51%) of the goats in the mammary tissue (Table 3). Only four goats had a doubtful PCR signal in milk (15%; CL: 1–29%); one from one udder halve, and three from both udder halves (Figure 3). All four of these goats had a PCR signal in both the intestines as in the mesenteric lymph nodes, although only one mammary tissue and none of the mammary lymph nodes reacted.

## 4. Discussion

The aim of this study was to determine the incidence of MAP DNA in colostrum and milk from dairy goats in infected herds, since the importance of MAP-contaminated colostrum and milk as a route of infection in dairy goat herds is unknown. The hypothesis as to whether or not MAP could be found in colostrum was answered as two out of the twenty-two samples of goats with clinical signs consistent with paratuberculosis were IS900 PCR-positive. The 120 colostrum samples (sample set I) were PCR-negative which was an unexpected outcome. Technical issues in the laboratory could be ruled out as a cause of the negative PCR results since there was repeated testing against control samples sets. Furthermore, in milk MAP, DNA was only detected in 4% of the samples of a cross section of a herd with a known paratuberculosis infection. A limitation of this is that it is a relatively low number of samples, and only from within one herd. For further research, this could be expanded.

In general, there are many knowledge gaps that hamper the prevention and control of MAP. Besides the fecal–oral route of transmission, transmission via inhalation of bio-aerosols has been shown in sheep and calves [23,24]. Separating kids at birth and rearing them separately reduces the environmental exposure risk. This is also indicated in dust sampling at twenty farms where all samples from the rearing areas were PCR-negative, as at ten of these Dutch dairy goat farms one or more, PCR-positive dust samples at the adult department and or milking parlor were found [25].

The possible risk for MAP transmission could be even further reduced by colostrum and/or milk treatment. Pasteurization by heating (high-temperature, long-time: 60 °C for 60 min, or high-temperature, short-time: 71.7 °C for 15 s, respectively) should be sufficient to eliminate MAP under most conditions from cow colostrum [26,27]. On-farm colostrum (cow and goat) pasteurization is gaining popularity within the Dutch dairy goat industry. A note should be made that colostrum handling and proper pasteurization, in regard to the temperature and time, is key, as IgGs should be preserved. Furthermore, cow colostrum inoculated with high MAP doses subjected to 10 kGy γ-irradiation, which is commonly used for sterilisation of foodstuffs and biological products, proved to be efficient in eliminating MAP without damage to immunoglobulin activity [28]. This method has been tried in Dutch dairy goats farms, but has been abandoned as taste is presumably affected and kids rejected it. Furthermore, transport of batches of deep-frozen colostrum to and from the operating facilities were deemed logistically challenging during busy kidding season. More recently, methods based on curdling and centrifugation were developed in Belgium [29]. These methods reduced MAP by 95%.

There seems to be an inconsistency between the ELISA and PCR results since there was no 100 percent one-on-one match between antibody and DNA positives in milk (sample set III) as shown in Table 2. Almost all of the 142 colostrum samples (sample set I + II) yielded a positive ELISA result with only two doubtful PCR results. However, antibody levels of colostrum were consistent with vaccination status, with vaccinated goats having a significantly higher colostrum antibody level, as was also demonstrated in a previous study [30]. It could be argued that goats from unvaccinated herds were less exposed as the perceived risk of paratuberculosis in those herds was lower. On the contrary, it is very likely that antibody levels in these unvaccinated herds derived from actual natural MAP exposure in the absence of vaccination titers. Since this is still a small proportion, it raises questions as to whether MAP pathobiology in goats is different from cattle and sheep, and whether or not MAP is present in the udder. Despite vaccination, 25 of the sacrificed goats with clinical signs had one or more IS900 PCR-positive tissue sample and therefore could be categorised as MAP-infected [25]. Although only 15% of the sacrificed MAP positive goats had a PCR signal in post mortem-collected milk, only one of these goats had a PCR signal in the mammary tissue. A positive PCR result indicates MAP DNA in the sampled material. It is, though, unclear in which fraction of the tissue this DNA is found. In the case of mammary tissue, MAP DNA was isolated from approximately three grams of homogenized material, consisting of glandular (potentially including milk) and connective tissue (potentially including blood vessel (and blood)). Both tissue and milk could harbor mononuclear phagocyte system cells and γδ T lymphocytes, which play a role in the (early) infection dynamics in cattle infected with MAP as well as free MAP. A PCR-positive result may identify both non-viable and viable bacteria, and in that sense, the current data may overestimate the risk with respect to the presence of infectious MAP in milk and colostrum. Proof of MAP viability was not included in this study design, but is suggested for further research.

In cattle colostrum and milk, contamination with MAP through fecal contamination of teats or shedding from within the udder is demonstrated [16,17,18]. A study in MAP-infected sheep indicated milk transmission from ewes to offspring and, moreover, detected MAP in the (macrophage cytoplasm of) mammary lymph nodes and glands [31] by an immunohybridization technique. In a Swiss study, 79 (23.0%, n = 344) goats’ tank-milk and 15 (23.8%, n = 63) ewes’ tank-milk samples were PCR-positive for MAP [32]. In Norway, MAP (immunomagnetic separation-PCR-dot blot hybridization) was detected in 24 (7.1%, n = 340) of individual goat milk samples. Furthermore, older vaccinated goats tested positive more often than does less than two years of age [33]. A Canadian study showed that bulk milk PCR testing had a poor sensitivity in both goats (0.0%) and dairy sheep (25.0%) although the ELISA performed better compared to fecal culture and PCR [34]. The results of that goat study may also indicate a low level of MAP shedding via milk, similar our results.

Judging MAP infection status by immune response-based serum test such as the ELISA has its limitations due to the substantial delay between infection and consistently positive serology [5]. Therefore, the sensitivity of the ELISA is limited in unvaccinated goats. Yet goats are seemingly the least naturally resistant to MAP, resulting in persistent fecal shedding, seroconversion, and clinical disease following MAP challenge [35]. Compared to other species this potentially attributes to an increased sensitivity of the ELISA and even so PCR. In the current study, the majority of goats was vaccinated with the non-DIVA Gudair ^®^ vaccine which precludes the use of the ELISA for determining the infection state. It has been recently shown that antibody levels post vaccination are influenced by age and environmental circumstances [25].

The transmission of MAP from dam to offspring by horizontal as well as vertical routes in MAP non-vaccinated cattle (major role: [36,37,38], minor role: [39]), goats, and sheep [31] has been researched in a number of studies. A meta-analysis in cattle quantifying age- and dose-dependence of early and late shedding of MAP indicated that challenging older calves or using multiple-exposure experimental systems resulted in a smaller proportion and shorter duration of early shedding as well as slower transition to late shedding from latent compartments [40]. Vice versa early exposure to a high dose of MAP resulted in more severe infections. Calves exposed naturally showed variable infection progression rates, not dissimilar to other infection routes [40]. So, feeding high load MAP-infected colostrum and/or milk to goat kids could potentially result in severe infections. A dam-daughter cohort study indicated that neither the presence of MAP DNA in colostrum, DNA in faeces, nor the presence of antibodies in colostrum significantly influenced the hazard of MAP shedding in their subsequent daughters up to the age of two years when raised in a contaminated environment [41]. Another study showed that heifer calves fed MAP DNA-positive colostrum were at no greater risk of MAP infection, compared with heifer calves fed MAP DNA-negative colostrum [42]. The same group suggested lack of evidence for fecal excretion of MAP in calves born to fecal culture positive (vs. negative) dams in a heavily infected herd [43]. These findings might very well apply to goat kids. Contamination from the surroundings (manure, dust) is a much more likely source of infection, also because MAP can survive well in the environment (soil and grass [44], dust [45]). Continous exposure and ingestion of MAP from environmental sources during rearing may contribute more to infection risk as compared to intake during the first day of life through colostrum. MAP non-vaccinated MAP shedders or MAP non-vaccinated does with a positive MAP antibody response at the beginning of their pregnancy were more likely to have an infected daughter positive to an IFN-gamma assay by the age of 15 months [30]. A study of a flock of MAP non-vaccinated MAP-infected sheep indicates both in utero and milk transmission of MAP from dams to their offspring [31]. These results in combination with the current findings suggest that MAP vaccination plays a role in limiting MAP transmission via colostrum and milk in goats.

## 5. Conclusions

The proportion of MAP DNA in colostrum and milk samples from MAP vaccinated goats originating from herds with paratuberculosis was lower than could be expected from cattle data, and the proportion of mammary tissue positives from goats with a clinical paratuberculosis infection, although antibodies (ELISA) were high (95% and 47% respectively). In conclusion, if the prevention of MAP infection is the only reason not to feed goat colostrum to kids, the benefits might outweigh the disadvantages as long as the herd is Map vaccinated and CAEV- and CL-certified free.

## Figures and Tables

**Table 1:** Background information of the dairy goat herds involved in the study on *Mycobacterium avium* subspecies *paratuberculosis* in dairy goat colostrum and milk.

Herd	Herd Size	MAP History	Age at MAP Vaccination	Sample Type	Age at Sampling (no. of Goats × Age in Years)	Lactation at Sampling (no. of Goats × Lactation)
A	840 Total 411 > 1 yo 429 < 1 yo	Known (post mortem)	At approx. 2 months	Colostrum	Colostrum: 10 × 1, 4 × 2, 1 × 3, 2 × 4, 1 × 5, 2 × 6.	10 × 1st, 5 × 2nd, 1 × 3rd, 3 × 4th, 1 × 5th.
B	962 Total 662 > 1 yo 300 < 1 yo	Known (post mortem and serology)	Between 1 to 8 months	Colostrum Milk Goats for post mortem	Colostrum: 6 × 1, 2 × 3, 2 × 4, 4 × 5, 2 × 6, 3 × 8. Mortem: 1 × 2, 1 × 5, 1 × 6, 1 × 8, 1 × 9.	6 × 1st, 6 × 2nd, 3 × 3rd, 5 × 4th.
C_nv_	1727 Total 1292 > 1 yo 435 < 1 yo	Unsuspected	Not applicable	Colostrum	Colostrum: 10 × 1.	10 × 1st
D	1673 Total 1287 > 1 yo 386 < 1 yo	Known (post mortem)	At approx. 3 months of age	Colostrum	Colostrum: 11 × 1, 8 × 2, 1 × 3.	19 × 1st, 1 × 2nd.
E	1533 Total 1153 > 1 yo 380 < 1 yo	Suspected	18 as goat kids 12 as adults	Colostrum	Colostrum: 8 × 1, 22 × 2.	30 × 1st
F_pv_	1226 Total 878 > 1 yo 348 < 1 yo	Known (serology)	Unknown	Colostrum	17 × no age data provided	17 × no lactation data provided
G	577 Total 453 > 1 yo 124 < 1 yo	Known (post mortem and serology)	Partly	Goats for post mortem	Post mortem: 1 × 4, 1 × 6, 1 × 9, 2 × 10, 1 × 11, 1 × 13.	Unknown
H	554 Total 435 > 1 yo 119 < 1 yo	Known (serology and post mortem)	Not applicable	Goats for post mortem	Post mortem: 1 × 2, 3 × 3, 1 × 4, 3 × 7, 1 × 9, and 6 × unknown.	Unknown
I	902 Total 676 > 1 yo 226 < 1 yo	Known (serology and post mortem)	As goat kids	Colostrum	22 × no age data provided	22 × no lactation data provided

**Table 2:** Distribution of ELISA (ID Vet MAP ELISA) and PCR (IS900) results in conveniently sampled milk samples of a dairy goat herd with a known *Mycobacterium avium* subspecies *paratuberculosis* infection.

	PCR Negative	PCR Dubieus	PCR Positive	Total
ELISA negative	102	2	2	106
ELISA dubious	22	0	1	23
ELISA positive	70	3	0	73
Total	194	5	3	202

**Table 3:** Distribution (1 = positive and 0 = negative) of PCR (IS900) results of intestines (distal ileum, mid jejunum, ileocecal valve), draining mesenteric lymph nodes as well as bilateral mammary tissue and mammary lymph nodes, and bilateral milk of goats designated to be slaughtered with clinical signs consistent with paratuberculosis.

	Pooled Tissue Samples				Milk L	Milk R	Number of Goats
Results Combination	Intestines	Mesenteric Lnn	Mammary Lnn	Mammary Tissue			
1	1	1	0	1	1	1	1
2	1	1	1	1	0	0	3
3	1	1	0	0	1	1	2
4	1	1	1	0	0	0	3
5	1	1	0	1	0	0	2
6	1	1	0	0	1	0	1
7	1	0	1	1	0	0	1
8	1	1	0	0	0	0	5
9	1	0	1	0	0	0	1
10	0	1	0	0	0	0	5
11	0	0	1	0	0	0	1
12	0	0	0	0	0	0	2
	Total: 19	Total: 22	Total: 9	Total: 7	Total: 4	Total: 3	Total no. of goats: 27
